# Diatom-Derived Polyunsaturated Aldehydes Activate Similar Cell Death Genes in Two Different Systems: Sea Urchin Embryos and Human Cells

**DOI:** 10.3390/ijms21155201

**Published:** 2020-07-22

**Authors:** Christian Galasso, Susanna Celentano, Maria Costantini, Salvatore D’Aniello, Adrianna Ianora, Clementina Sansone, Giovanna Romano

**Affiliations:** 1 Department of Marine Biotechnology, Stazione Zoologica Anton Dohrn, 80121 Naples, Italy; susanna.celentano@szn.it (S.C.); maria.costantini@szn.it (M.C.); ianora@szn.it (A.I.); giovanna.romano@szn.it (G.R.); 2 Department of Biology and Evolution of Marine Organisms, Stazione Zoologica Anton Dohrn, 80121 Naples, Italy; salvatore.daniello@szn.it

**Keywords:** apoptosis, autophagy, human cells, sea urchin, *Paracentrotus lividus*, cell death pathways, marine compounds, conserved cell death

## Abstract

Programmed cell death, such as apoptosis and autophagy, are key processes that are activated early on during development, leading to remodelling in embryos and homeostasis in adult organisms. Genomic conservation of death factors has been largely investigated in the animal and plant kingdoms. In this study, we analysed, for the first time, the expression profile of 11 genes involved in apoptosis (extrinsic and intrinsic pathways) and autophagy in sea urchin *Paracentrotus lividus* embryos exposed to antiproliferative polyunsaturated aldehydes (PUAs), and we compared these results with those obtained on the human cell line A549 treated with the same molecules. We found that sea urchins and human cells activated, at the gene level, a similar cell death response to these compounds. Despite the evolutionary distance between sea urchins and humans, we observed that the activation of apoptotic and autophagic genes in response to cytotoxic compounds is a conserved process. These results give first insight on death mechanisms of *P. lividus* death mechanisms, also providing additional information for the use of this marine organism as a useful in vitro model for the study of cell death signalling pathways activated in response to chemical compounds.

## 1. Introduction

Programmed cell death (PCD) is a fundamental cellular process regulated by genomic information preserved during evolution that occurs in both animal and plant kingdoms. PCD is activated by a plethora of stimuli and is regulated by many signal transduction pathways, which leads to gene-controlled death in damaged cells that are, therein, eliminated from tissues [1]. Death processes can also occur in “healthy” cells, such as during differentiation and development [2], for cell turnover and immune system regulation [3,4]. Death processes activated during embryonic development play a crucial role for the rearrangement and shaping of germ layers and, consequentially, for the formation of anatomical structures [5]. Molecular and morphological features of several types of PCD have been well characterised. The most common morphological and biochemical alterations induced by PCD include cell contraction, formation of membrane blebs, and cleavage of specific caspases that elicit enzymatic breakdown of DNA [6].

Among different types of PCD, apoptosis and autophagy are considered the most evolutionary conserved death processes across the metazoan lineage [7]. Autophagy is a catabolic process implicated in the degradation of dysfunctional organelles for the correct cell homeostasis, while apoptosis removes transformed or damaged cells, preventing their aberrant proliferation [8]. These tightly correlated cell death pathways also act in synergy in non-physiological conditions, such as in cancer onset and progression, where they represent defence mechanisms to control cancer development. In fact, apoptosis and autophagy are implicated in the inhibition of oncogenesis through the activation of death signalling in cells with altered homeostatic equilibrium, which represents the starting point for pre-neoplastic lesions. Many regulatory factors control both death pathways, including the pro-apoptotic factor p53 [9], B-cell CLL/lymphoma 2 (BCL2) family members, Fas-associating death domain-containing protein (FADD), and some autophagy-related (ATG) proteins [10].

Apoptosis and autophagy have been extensively studied in several vertebrates, such as *Danio rerio* (zebrafish), and invertebrates, such as *Drosophila melanogaster* (fruit fly) and *Caenorhabditis elegans* (worm) [11,12,13]. There is also evidence of in vitro activation of autophagy and apoptosis on human cancer cell lines after exposure to marine compounds [14,15,16]. These studies have allowed the identification of potential molecular targets for chemoprevention and chemotherapy [17].

The sea urchin is an already well-established marine model organism for developmental biology studies, largely used in drug discovery research for the understanding of cell proliferation processes induced by natural compounds with antimitotic activity [18,19]. In the last two decades, many studies have demonstrated the activation, at the enzymatic and morphological level, of apoptosis in sea urchins [20,21,22,23]. Other studies have focused on differences in expression levels of genes related to teratogenic processes and stress responses in sea urchins exposed to toxic marine compounds [24,25,26]. Moreover, sea urchins have been used for the assessment of the effect of inorganic and organic pollutants [27].

In our previous study, we found 11 death-related genes of the sea urchin *Paracentrotus lividus*, orthologs of the human death-related factors, which are known to be involved in autophagy, extrinsic apoptosis, and intrinsic apoptosis [28]. On the basis of these results, in the present study, we analysed, for the first time, gene expression variation of the 11 death-related genes in *P. lividus* embryos exposed to three antiproliferative marine compounds. Moreover, we compared the expression profiles of these 11 sea urchin genes in embryos with the 11 human orthologs in A549 cells (adenocarcinomic alveolar basal epithelial cells) exposed to same compounds. The rationale of this work was to describe the genes target and response of *P*. *lividus* to three diatom-derived polyunsaturated aldehydes (PUAs) and to demonstrate conservation, in human cells, of death-related response to the same marine compounds [22,29]. We focused our attention on the dominant bioactive PUAs released by diatoms, namely, the C10 2-trans-4-trans-decadienal (from now on, decadienal), the C8 2-trans-4-trans-7-octadienal (from now on, octadienal), and the C7 2-trans-4-trans-7-heptadienal (from now on, heptadienal). These compounds belong to the oxylipin family, low molecular weight secondary metabolites produced by plants and animals as well as by some species of diatoms, a major class of marine microalgae. They are not constitutively present in diatoms, but are produced only when cells are damaged, as would occur during grazing by copepods, or released at the end of blooms by senescent cells undergoing to lysis. PUA production is initiated by lipase enzymes catalysing the release of fatty acid precursors from cell membranes, which in turn are oxidised by lipoxygenase enzymes and cleaved by hydroperoxide lyases [30]. PUA biological activity has been extensively studied [31], and their role and chemical interactions in planktonic and benthic communities are well documented [32,33]. Nevertheless, only recently investigations focusing on molecular mechanisms have been reported [25,26]. Some studies demonstrated that these aldehydes inhibit cell cleavage and induce strong teratogenic effects on embryonic cells (i.e., actively proliferating cells) during larvae development in different marine organisms [34,35], without toxic effects on adults (i.e., somatic cells) [36]. A comparable effect was observed on human cell lines, where PUAs act as antiproliferative compounds on cancer cells, exhibiting specific effects on A549 and COLO205 (human colorectal adenocarcinoma), without affecting normal cell viability (BEAS-2B, derived from bronchial epithelium) [29]. The teratogenic effect of diatom-derived PUAs has been linked to the impairment of developmental process and the induction of apoptosis, as revealed by fluorescent staining techniques [22,23]. In the present study, we aimed at elucidating, at a gene level, the death response activated in response to PUAs in the sea urchin *P*. *lividus*, showing the occurrence of similar gene expression profile in human cells, notwithstanding the evolutionary distance between the two model systems.

## 2. Results

### 2.1. Morphological Analysis of Sea Urchin Larvae and A549 Cells

At 48 h post-fertilisation (hpf), larvae treated with the three PUAs exhibited several types of abnormalities, comprising delayed embryos (morula, blastula, gastrula, or prism stage) and malformed plutei (with crossed or separated tip, fused or not fully developed arms, and incomplete skeletal rods) (see Figure S1). Percentage of abnormal larvae treated at five increasing PUA concentrations are reported in Table 1. These results illustrate the dose-dependent effect of PUAs on sea urchin embryonic development. Percentage (%) of abnormality ranged from 14.7 to 74.9 after decadienal treatment, from 15.6 to 73.8 after heptadienal treatment, and from 12.1 to 76.8 after octadienal treatment. Controls exhibited low percentage of abnormal larvae (≤5.4%). Sea urchin embryos were also observed at 5 and 21 hpf, verifying normal development in controls and abnormalities in treated groups (see Figure S2). In particular, embryos treated at intermediate and high doses of PUAs were delayed at 5 and 21 hpf and presented alterations in the formation of blastocoel (at 5 hpf) and in the three-layered body plan and larval skeleton (at 21 hpf). Controls (at 5 and 21 hpf) presented normal morphology and development.

A549 cells without treatments appeared with green fluorescence, since acridine orange was able to penetrate in living cells, intercalating into intact double-strain nucleic acids. On the contrary, cells treated with the three PUAs presented double staining due to alteration of cell membrane permeability (red fluorescence of ethidium bromide) and presence of broken single strand nucleic acids (red fluorescence of acridine orange). Moreover, irregular cell contour was observed in the A549 treated with PUAs (see Figure S3).

### 2.2. Effect of Decadienal on Gene Expression of Sea Urchins and Human Cells

**Figure 1:**
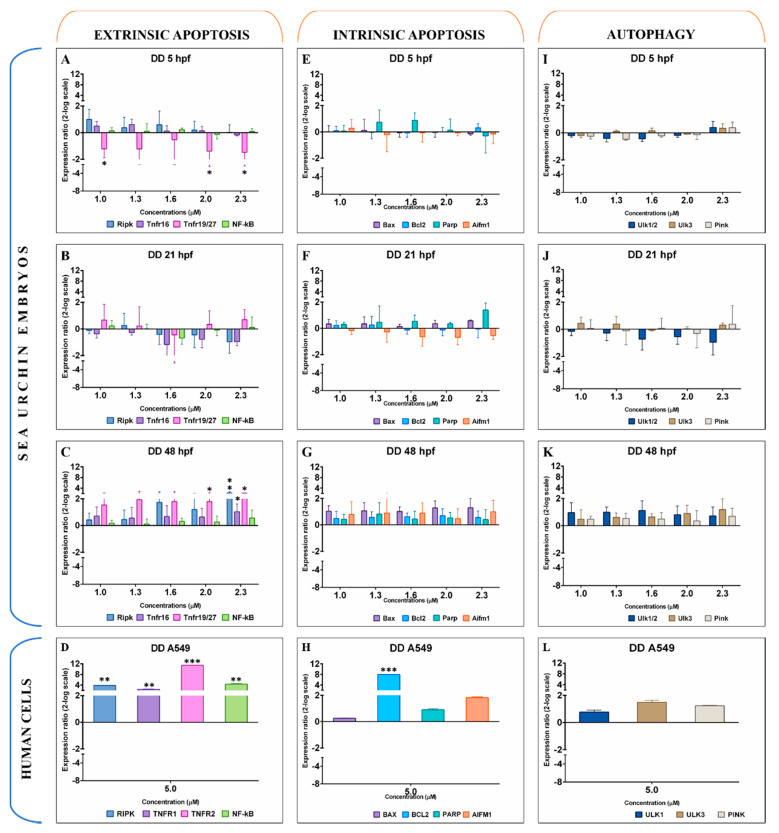
Gene expression levels of 11 genes belonging to extrinsic apoptosis (**A**–**D**), intrinsic apoptosis (**E**–**H**), and autophagy (**I**–**L**) in the sea urchin *Paracentrotus lividus* treated with 1.0, 1.3, 1.6, 2.0, and 2.3 μM of decadienal for 5, 21, and 48 hpf and in A549 human cells treated with 5 μM for 2 h. Samples obtained from three independent biological replicates were analysed by qPCR in technical triplicates. Data are reported as fold difference (mean ± SD), compared to controls without decadienal. Error bars represent the variation in biological replicates. Expression values were statistically analysed by Mann–Whitney *U* test, and values were considered significant if the *p*-value was ≤0.05 (* for *p*-value ≤ 0.05; ** for *p*-value ≤ 0.01; *** for *p*-value ≤ 0.001).

Decadienal did not significantly affect expression levels of any of the genes studied in sea urchin embryos at 5 and 21 hpf (Figure 1A,B,E,F,I,J), with the only exception being *Tnfr19/27* (Tumour necrosis factor receptor 19/27) that was significantly downregulated at low (1.0 μM) and high (2.0 and 2.3 μM) concentrations after 5 hpf (Figure 1A). Three genes involved in the extrinsic apoptosis were upregulated at 48 hpf: *Ripk* (Receptor-interacting serine-threonine kinase) and *Tnfr16* at 2.3 μM (2.2 and 1.79 log2-fold change, hereinafter abbreviated as log2FC), and *Tnfr19/27* at 1.3 and 2.3 μM (2.0 and 2.2 log2FC, respectively) (Figure 1C). A549 cells activated all genes involved in extrinsic apoptosis after exposure to 5.0 μM of decadienal (Figure 1D). In particular, decadienal upregulated receptors implicated in this specific pathway (such as *TNFR1* and *TNFR2*, 2.4 and 11.4 log2FC) and down-streams factors (such as *RIPK2* 3.9 and *NF-κB*-Nuclear factor kappa B-, and 4.4 log2FC). *BCL2* (7.9 log2FC) was the only intrinsic apoptotic gene upregulated in A549 by decadienal (Figure 1H), but not in the sea urchin embryos at all developmental stages examined (5, 21, and 48 hpf, Figure 1E–G). Autophagic genes were not found significantly up- or downregulated in both embryos and A549 cells (Figure 1I–L).

### 2.3. Effect of Heptadienal on Gene Expression of Sea Urchins and Human Cells

**Figure 2:**
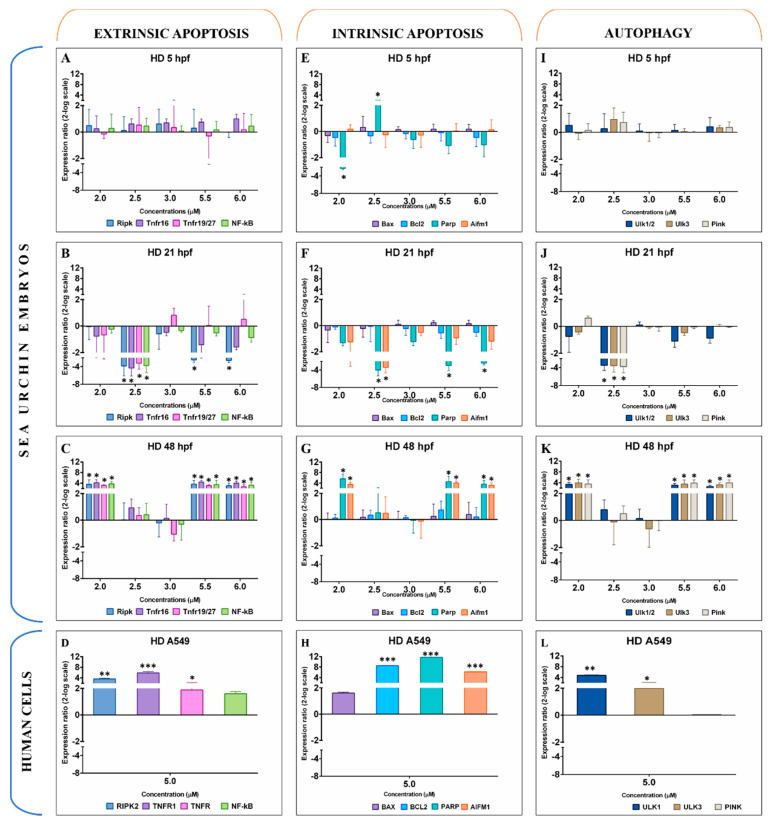
Gene expression levels of 11 genes belonging to extrinsic apoptosis (**A**–**D**), intrinsic apoptosis (**E**–**H**), and autophagy (**I**–**L**) in the sea urchin *Paracentrotus lividus* treated with 2.0, 2.5, 3.0, 5.5, and 6.0 μM of heptadienal for 5, 21, and 48 hpf and in A549 human cells treated with 5.0 μM for 2 h. Samples obtained from three independent biological replicates were analysed by qPCR in technical triplicates. Data are reported as fold difference (mean ± SD), compared to controls without heptadienal. Error bars represent the variation in biological replicates. Expression values were statistically analysed by Mann–Whitney *U* test and values were considered significant if the *p*-value was ≤0.05 (* for *p*-value ≤ 0.05; ** for *p*-value ≤ 0.01; *** for *p*-value ≤ 0.001).

Sea urchin embryos exposed to heptadienal did not show variation in gene expression at 5 hpf for the majority of genes studied (Figure 2A,E,I). Only *Parp* (Poly ADP-ribose polymerase) was downregulated at 2.0 μM and upregulated at 2.5 μM (−2.3 and 2.0 log2FC; Figure 2E). All genes were found downregulated at 21 hpf. Extrinsic apoptotic genes *Ripk*, *Tnfr16*, *Tnfr19/27*, and *NF-κB* were downregulated by 2.5 μM of heptadienal (−3.8, −4.3, −3.2, and −3.8 log2FC, respectively); *Ripk* remained downregulated also at 5.5 and 6 μM (Figure 2B). *Parp* and *Aifm1* (Apoptosis-inducing factor, mitochondrion-associated, 1) were downregulated at 2.5 μM (−4.0 and −3.4 log2FC), with *Parp* remaining significantly downregulated at the two highest concentrations also (−2.9 and −2.4 log2FC). Autophagic genes (Unc-51-like kinases 1/2 and 3 and PTEN induced putative kinase) were found downregulated at 2.5 μM (*Ulk1/2*, *Ulk3*, and *Pink* at −3.5, −3.6, and −3.8 log2FC, respectively; Figure 2J). Heptadienal induced upregulation of all genes studied at 48 hpf, except for *Bax* (BCL2-associated X protein) and *Bcl2*. In particular, extrinsic genes were upregulated at 2.0, 5.5, and 6.0 μM (Figure 2C), such as *Ripk* (3.5, 3.7, and 3.0 log2FC), *Tnfr16* (4.2, 4.4, and 4.0 log2FC), *Tnfr19/27* (3.2, 3.1, and 3.3 log2FC), and *NF-κB* (3.7, 3.5, and 3.2 log2FC). Intrinsic apoptotic genes *Parp* and *Aifm1* were upregulated by 2.0, 5.5, and 6.0 μM (5.6, 4.6, and 3.6 log2FC, respectively, for *Parp*; 3.5, 4.0, and 3.2 log2FC, respectively, for *Aifm1*). A similar expression profile was observed also for autophagic genes at 48 hpf (Figure 2K), with upregulation of *Ulk1/2*, *Ulk3*, and *Pink* at 2.0 μM (3.4, 3.9, and 3.5 log2FC, respectively), 5.5 μM (3.1, 3.6, and 3.9 log2FC, respectively), and 6.0 μM (2.6, 3.3, and 4.0 log2FC, respectively). In A549 cells, heptadienal upregulated all extrinsic apoptosis genes (*RIPK2*, *TNFR1*, *TNFR2*, and *NF-κB* at 3.1, 8.1, 4.5, and 3.4 log2FC, respectively). With the exception of *BAX*, all intrinsic apoptotic genes were also upregulated (*BCL2*, *PARP*, and *AIFM1* at 8.5, 11.7, and 6.2 log2FC, respectively). *ULK* autophagic genes were upregulated with 5.0 μM of heptadienal (*ULK1* and *ULK3* at 4.8 and 2.0 log2FC, respectively).

### 2.4. Effect of Octadienal on Gene Expression of Sea Urchins and Human Cells

**Figure 3:**
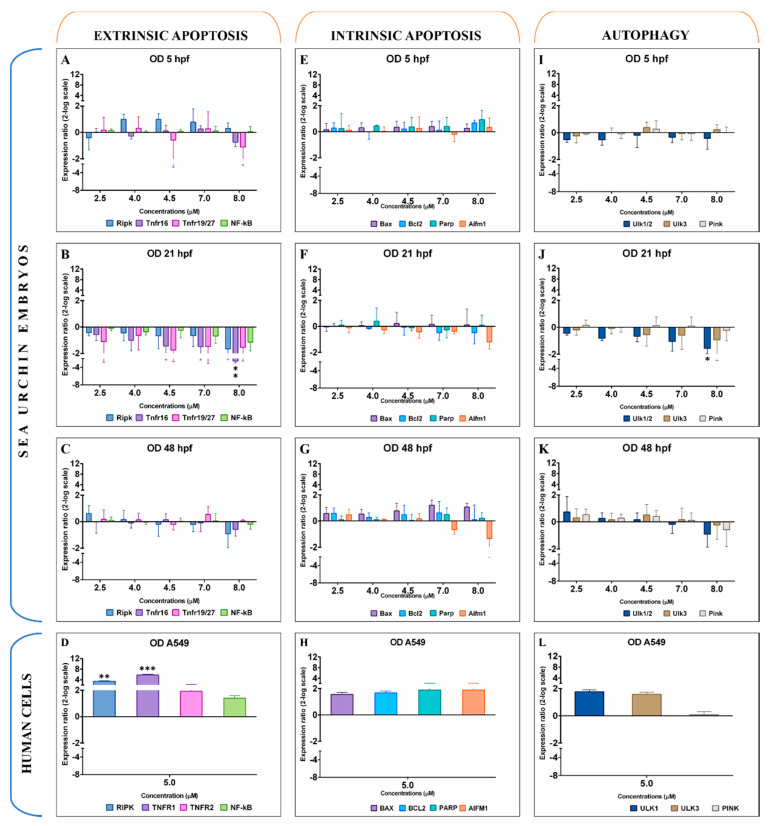
Gene expression levels of 11 genes belonging to extrinsic apoptosis (**A**–**D**), intrinsic apoptosis (**E**–**H**), and autophagy (**I**–**L**) in the sea urchin *Paracentrotus lividus* treated with 2.5, 4.0, 4.5, 7.0, and 8.0 μM of octadienal for 5, 21, and 48 hpf and in A549 human cells treated with 5.0 μM for 2 h. Samples obtained from three independent biological replicates were analysed by qPCR in technical triplicates. Data are reported as fold difference (mean ± SD), compared to controls without octadienal. Error bars represent the variation in biological replicates. Expression values greater were statistically analysed by Mann–Whitney *U* test and values were considered significant if the *p*-value was ≤0.05 (* for *p*-value ≤ 0.05; ** for *p*-value ≤ 0.01; *** for *p*-value ≤ 0.001).

Octadienal did not significantly influence gene expression levels after 5 hpf in embryos (Figure 3A,E,I). Embryos treated with 8.0 μM of octadienal showed downregulation of *Tnfr16* and *Ulk1/2* at 21 hpf (−2.5 and −1.7 log2FC) (Figure 3B). After 48 hpf, octadienal did not induce any effect on gene expression levels (Figure 3C,G,K). Similar results were obtained on A549 cells, where octadienal did not up- or downregulate genes involved in intrinsic apoptosis and autophagy (Figure 3H,L). On the contrary, two extrinsic apoptosis genes were upregulated with 5.0 μM of octadienal (*RIPK2* and *TNFR1* at 3.3 and 5.9 log2FC, respectively; Figure 3D).

## 3. Discussion

This work investigated, for the first time, the expression profile of 11 cell death-related genes in embryos of the sea urchin *P. lividus* exposed to three PUAs, focusing on key factors involved in extrinsic and intrinsic apoptosis, as well as autophagy. The gene expression profile was compared with that obtained after treatment of human adenocarcinoma A549 cells with the same three molecules. We compared death response of these two models since cancer and embryonic cells are actively proliferating cells with high growth rate that have already shown growth inhibition when treated with PUAs, conversely to their normal counterparts (adult sea urchins and normal human cells) [29,36].

Morphological analysis on *P*. *lividus* was performed on plutei, since morphological features are well-defined at 48 hpf, to validate the range of concentrations chosen for each PUA, in order to obtain a comparable effect at the phenotypical level among the three molecules (Figure S1). Embryo development was also observed at 5 and 21 hpf, showing alterations and delays with respect to the controls (Figure S2). The morphological effects we observed are in accordance with previous findings [25]. Indeed, PUAs acted in a dose-dependent manner, inducing abnormalities and delay in sea urchin development in 15–75% of plutei (larval stage) when embryos were treated with increasing concentrations. Decadienal, heptadienal, and octadienal provoked same type of malformations, such as separated and crossed tips, and plutei with not fully developed arms and with incomplete skeletal rods (Figure S1). Morphological analysis was also performed on human cells, demonstrating activation of cell death process after PUA treatment. In particular, A549 cells presented double staining (acridine orange and ethidium bromide), changing in cell structure, nuclear fragmentation, and loss in membrane integrity (Figure S3); these cell features reveal triggering of cell death pathways in the cells. PUAs concentrations higher than those used in this work were able to induce strong antiproliferative effects and cleavage arrest in sea urchin embryos [37]. Romano and collaborators identified concentrations of decadienal, heptadienal, and octadienal provoking cleavage inhibition. In particular, the arrest at first cleavage of sea urchin *P*. *lividus* embryos occurred at 5.26 μM of decadienal, 27.27 μM of heptadienal, and 16.13 μM of octadienal. Activation of apoptotic process was revealed by means of the Terminal deoxynucleotidyl transferase dUTP nick end labeling (TUNEL) assay on plutei at 48 hpf. In addition, Ruocco and colleagues showed caspase activation using specific fluorescent substrate for caspase 3/7 and caspase 8 [36]. Sansone and colleagues tested PUAs on the A549 cell line at 10.0 µM, showing a strong cytotoxic effect (cell viability lower than 30%), with severe intracellular alterations and very early cell death activation, while 5.0 µM provoked moderate reduction of viability and activation of many death factors [29]. Thus, concentrations used in the present work were chosen to induce in embryos and A549 a moderate cell death stimulation, which gave clear gene expression profiles.

**Figure 4:**
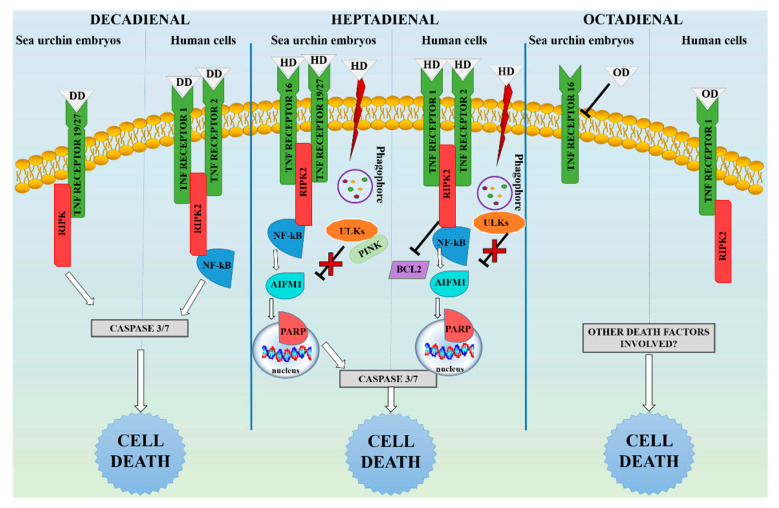
Schematic representation of death genes activated in the sea urchin *Paracentrotus lividus* embryos and A549 human cells. All genes studied by qPCR and significantly upregulated or downregulated by PUAs are represented with a specific colour, whereas caspase 3/7 (represented in a grey rectangle) was already investigated in a previous study [23] and was used in this figure only to complete the pathway. Phagophores engulf cytoplasmic components during autophagy that are delivered to lysosomes for degradation. “DD” refers to decadienal, “HD” refers to heptadienal, and “OD” refers to octadienal.

Decadienal induced in embryos and human cells a similar expression profile of genes involved in the extrinsic apoptotic pathways (Table 2 and Figure 4). After 48 hpf, sea urchin embryos showed an upregulation of genes encoding for death receptors (*Tnfr 16* and *Tnfr 19/27*) and an intracellular effector (*Ripk*), which are involved in the extrinsic apoptosis. Moreover, the same PUA was able to upregulate *Aifm1* and *Ulk3*, indicating induction of mitochondrial damage [38,39]. Decadienal, before larval stage (at 5 and 21 hpf), did not provoke significant variation of expression levels in all genes tested, with the only exception being the downregulation of *Tnfr19/27* at 5 hpf. These results indicate that receptor-mediated apoptosis was not the mechanism used by sea urchin to respond to chemicals in the first stages of embryo development, in agreement with previous findings [40]. A similar gene expression profile was detected in human cells, where all upstream (*TNFR1* and *TNFR2*) and downstream (*RIPK2* and *NF-κB*) factors were significantly upregulated, indicating an extrinsic apoptotic response to decadienal treatment. The other two cell death pathways investigated did not seem to be affected by decadienal in A549 cells. With regard to intrinsic apoptotic and autophagic genes, *BCL2* was the only gene found to be significantly upregulated in human cells. Upregulation of *BCL2* was the only difference in the response induced by decadienal between sea urchin embryos and A549 expression profiles. This is not in contrast with activation of the extrinsic apoptosis, since the *BCL2* gene encodes for a regulator of cell death that modulates mitochondrial membrane permeability; its upregulation is linked with the inactivation of the intrinsic (mitochondria-dependent) pathway of apoptosis, favouring other death mechanisms [41].

In sea urchin embryos, the variation in gene expression showed an absence of linear dose–response relationship, especially with heptadienal. In fact, low and high concentrations induced significant a variation of gene expression, while intermediate dose of heptadienal (i.e., 2.5 and 3.0 µM) did not affect expression of the same genes. Sea urchin embryos treated with the same molecules showed this unconventional dose–response profile related to other processes, such as skeletogenesis, differentiation, detoxification, and stress-related response [42]. *P*. *lividus* genes such as DNA-methyltransferase, Sex-determining Region Y (SRY)-box9, p38 mitogen-activated protein (MAP) kinase, heat shock protein 56, multi-drug resistance protein 1, and cytochrome b exhibited variation in transcriptional levels only at low and high doses of decadienal and heptadienal. This unconventional expression profile reported by Varrella and colleagues [42] and in the present work, even if obtained on different genes and different pathways, seem to have a common behaviour, probably linked to biological activity and chemical features of this class of compounds. Since at the morphological level we observed an almost linear dose–response effect on malformation appearance, the response at the gene level may reflect the involvement of different signalling pathways affecting a common endpoint with opposing effects. This complexity may lead to the overlapping and integration of two or more monotonic dose–response curves, resulting in an atypical dose–response effect [43]. Another possible explanation comes from a study by Gualtieri and colleagues, who demonstrated that Sertoli cells exposed to various doses of bisphenol A (0.5 nM–100 μM) undergo an increase in cell protection by higher glutathione levels only at intermediate doses (10 μM–50 μM), not at high or low doses [44]. They showed that detoxification was enhanced at intermediate levels, while cell viability was negatively affected at high and low doses since the cells were incapable of eliciting a defensive response mechanism at these doses. Our results suggest that sea urchin embryos respond to intermediate concentrations of heptadienal, activating at 48 hpf survival and recovery mechanisms, while at low or high doses of the compound, it may trigger cell death. In addition, heptadienal downregulated most of the genes studied after 21 hpf in sea urchins. During gastrulation, embryos undergo extensive cell rearrangement, using death mechanisms for appropriate formation of body structures [45]. Heptadienal probably interfered with or delayed death processes that occur during the rearrangement of normal development (e.g., apoptosis and autophagy). These genes were found to be highly expressed at 21 hpf in normal conditions [28], and thus embryonic perturbation and delay induced by heptadienal at 21 hpf (Figure S2) may cause the reduction of gene expression with respect to control level (Figure 2B,F,J). The subsequent activation of death genes at the larval stage (48 hpf) suggests that embryos can tolerate and detoxify the harmful effects of heptadienal until accumulation of cell damage and cytotoxicity, which produces an overload on the defence system that is unable to adequately respond, culminating in the activation of cell death [46]. Heptadienal induced a complex gene response in sea urchin embryos and human cells (Table 2 and Figure 4) and did not seem to specifically act on a single death mechanism, upregulating at 48 hpf several factors involved in all death mechanisms investigated. As for decadienal, overlapping profiles of gene expression between *P*. *lividus* and human cells were observed also with heptadienal. Data obtained in both experimental models (at 48 hpf for embryos and 2 h for A549) suggest a predominant participation of extrinsic apoptotic genes and an involvement of autophagic factors (Table 2). In fact, heptadienal induced in embryos the upregulation of all genes involved in extrinsic apoptosis (*Tnf* receptors and intracellular effectors, such as *Ripk* and *NF-κB*) and activation of *Parp*, *Aifm1*, *Pink*, and *Ulks* genes. *Aifm1* encode for a mitochondrial-associated protein involved in DNA fragmentation processes, which lead to cell death [47,48]. *PARP* is a nuclear factor connected to DNA damage and is activated by caspase 3, which represents the intersection between intrinsic and extrinsic apoptosis [49,50]. The hypothesis of heptadienal-mediated mitochondrial damage is also supported by activation of all autophagic factors (*Ulk1/2*, *Ulk3*, and *Pink*). These genes are activated when cells organise a selective clearance of damaged mitochondria [51]. On the other hand, in human cells, all extrinsic factors and *BCL2* (negative regulator of intrinsic apoptosis) were upregulated; thus, heptadienal can induce an extrinsic apoptotic response and inactivation of the intrinsic pathway. Heptadienal activated autophagic factors also in human cells (*ULK1* and *3*), which may indicate an attempt of cells to remove chemical injury by removing damaged intracellular organelles. For this reason, the death mechanism involved, at the gene level, in response to heptadienal seemed to be, in both models, apoptosis via the extrinsic pathway, with the involvement of nuclear and mitochondrial factors. Autophagy contributes to maintenance of cellular homeostasis. It regulates elimination and recycling of damaged intracellular compartments, also providing energy (ATP) for the apoptotic machinery [52]. Thus, human cells and embryos can tolerate chemical injury (e.g., heptadienal), trying to reduce its harmful effects and damages. Autophagy activates survival or death signals on the basis of the entity of cell damage. In our case, heptadienal seemed to cause, after 48 hpf, accumulation and irreversible cellular damages that cannot be recovered by autophagic or other cellular defence processes, inducing activation of cell death. Moreover, heptadienal upregulated *Pink* only in sea urchin embryos. Pink is a key factor of mitophagy, a selective degradation mechanism of mitochondria, which may be connected with the activation of those embryonic processes that lead to the elimination of damaged mitochondria during *P*. *lividus* development [53,54].

Octadienal was the least toxic PUA tested (Table 2 and Figure 4), as also demonstrated by Sansone et al. [29]. In particular, none of the genes analysed were targeted by this PUA in *P*. *lividus*, since the only effect observed on gene expression was a downregulation of *Tnfr16* at the highest concentrations tested (8.0 μM after 21 hpf). Similar results were found in human cells, with octadienal having no effect on gene expression of apoptotic and autophagic factors, with the exception of *TNFR1* and *RIPK*. The activation of those two genes is probably not sufficient to hypothesise activation of a specific cell death pathway in humans. In fact, they are involved also in many other cellular mechanisms, such as necroptosis and inflammation [55,56].

Our results showed, at the gene level, comparable cell death response to PUAs in the two evolutionary distant organisms investigated. Many studies have demonstrated the genomic conservation of death genes between humans and evolutionarily distant organisms [28,57,58], but few works have attempted to investigate molecular response conservation after exposure to the same stimuli [59]. Our study offers a first piece of evidence that apoptosis and autophagy could be connected processes in the death-related gene response to PUAs. These pathways are conserved during evolution, not only in terms of genomic information but also in terms of functional role, by involving the same gene cascades as response to chemical treatments, leading to PCD. Further studies to assess the induction of cell death pathways at the protein level and spatial expression of these death-related factors in developing embryos are necessary to better clarify the activation of apoptosis and autophagy in these in vitro systems after exposure to PUAs.

Altered pathways involved in cell survival and death cause many human pathologies. Sea urchin embryos have been employed as model system to study molecular mechanisms behind human pathologies, including neurodegenerative and cancer diseases [60]. This experimental work identified a gene panel that could be useful to study activation of cell death in *P*. *lividus*, although a larger number of genes is required to investigate a wider array of cell death mechanisms.

These results also suggest that sea urchins could be used as a model organism to study, at the gene level, death mechanisms in response to environmental pollutants and toxic compounds, as well as for the preliminary screening of new natural compounds with antiproliferative and cytotoxic effects, useful for the pharmaceutical research sector.

## 4. Materials and Methods

### 4.1. Harvesting of Animals, Embryo Treatments, and Morphological Analysis

Sea urchin *P. lividus* specimens were harvested in the Gulf of Naples. Animals were maintained in large tanks with sea water for at least 5 days to allow acclimatization. Gametes were obtained by injection of 0.5 M of potassium chloride (KCl) in the adult animals. Eggs were dispensed in crystallizing dishes: 160 eggs mL^−1^, in a final volume of 50 mL of filtered sea water (FSW), for a total of 8000 eggs per experiment. Each treatment was carried out in biological triplicate, using eggs from three different females, fertilized with a mix of sperm collected from 4–5 males. Sea urchin embryos without any treatment were allowed to develop for 5, 21, and 48 hpf and used as controls. PUAs dissolved in methanol (≤0.1% final concentration) were used to treat eggs at the following PUA concentrations: 1.0, 1.3, 1.6, 2.0, and 2.3 μM of decadienal (CAS number 25152-84-5); with 2.0, 2.5, 3.0, 5.5, and 6.0 μM of heptadienal (CAS number 4313-03-5); with 2.0, 4.0, 4.5, 7.0, and 8.0 μM of octadienal (CAS number 5577-44-6) (Sigma-Aldrich, St. Louis, MO, USA). These concentrations were chosen since they create a wide range of embryonic perturbation and severity, from mild to severe abnormality, and delay [25]. Ten minutes after PUA treatment, eggs were fertilized with 180 μL of diluted sperm (1:1000 sperm/FSW) and embryos were then incubated for 5, 21, and 48 hpf in a thermostatic chamber at 19 °C with a 12:12 h light/dark cycle. After each incubation time, embryos were centrifuged at 3600 rcf (relative centrifuge force) for 15 min at 4 °C. Pellets were stored at −80 °C for gene expression study. At 5, 21, and 48 hpf, an aliquot (1 mL) of larval cultures were observed with inverted microscope to evaluate morphology. Pluteus larvae were chosen for the assessment of abnormalities. Larvae that showed crossed or separated spicules, fused or asymmetrical arms, or developmental delay were considered abnormal.

### 4.2. RNA Extraction and cDNA Synthesis from Paracentrotus lividus Embryos

TRIsureTM reagent (1 mL) was added to frozen pellets of *P. lividus* embryos for RNA extraction, according to the manufacturer’s instructions (Bioline, London, UK, cat. no. BIO-38032). Direct-zol RNA MiniPrep (Zimo Research, Irvine, CA, USA, cat. no. R2051) was used to isolate total RNA by spin column. gDNA (genomic DNA) was degraded by using a DNase RNase-free kit (Roche, Mannheim, Germany, cat. no. 4716728001), according to the manufacturer’s instructions. RNA samples were run on gel (agarose 1%, ethidium bromide 0.5 μg mL^−1^) for the evaluation of integrity (evaluating 18S and 28S bands). RNA samples were reverse-transcribed using an iScript cDNA (complementary DNA) Synthesis kit (Biorad, Hercules, CA, cat. no. 1708891), following the manufacturer’s instructions, to obtain cDNA.

### 4.3. qPCR for Gene Expression Analysis on Sea Urchin Embryos

Quantitative PCR (qPCR) experiments were run to evaluate variations of gene expression in three stages of *P. lividus* development (blastula, at 5 hpf; gastrula, at 21 hpf; pluteus larvae, at 48 hpf). qPCR experiments were set up using cDNAs (5 ng μL^−1^, final concentration) with 0.3 μM of forward and reverse primers and 1 × Fast SYBR Green Master Mix (Applied Biosystems, Austin, TX, USA, cat. no. 4385612), in a total volume of 10 μL. Primers of investigated genes were illustrated in Table 3 (Coding DNA Sequences and amplicons were reported in the supplementary material of Galasso et al. [28]). Melting curve analysis was performed for each primer pair. The efficiency of each primer pair was calculated using the equation E = 10^−1/slope^. Five serial dilutions were set up to determine Ct values (1:5, 1:10, 1:50, and 1:100). PCR efficiencies were calculated for control and target genes, and were found between 1.9 and 2.

Amplifications were run in a ViiATM7 Real-Time PCR System (Applied Biosystems) thermal cycler. The first stage was at 95 °C for 20 s; the second stage was composed by 40 cycles at 95 °C for 1 s and 60 °C for 20 s; the final stage (melt curve) was at 95 °C for 15 s, 60 °C for 1 min, and 95 °C for 15 s. qPCR reactions were performed in triplicate, and each experiment included a no-template control for each primer pair. The expression of each gene was analysed using REST software (Relative Expression Software Tool, available online at https://www.gene-quantification.de/rest.html) based on the Pfaffl method [62,63]. qPCR results were normalized using *Pl-Z12-1* gene (zinc-finger transcription factor) as reference, since its expression is almost constant in all the developmental stages examined (5, 21, and 48 hpf) and after PUA treatments (Figure S4; as also shown in Costa et al. [64]).

### 4.4. A549 Cell Culture, Treatment with PUAs, Morphological Analysis, and qPCR

The adenocarcinomic human alveolar basal epithelial cell line A549 was purchased from the American Type Culture Collection (Manassas, VA, USA, ATCC CCL185). Cells were maintained in appropriated medium (Dulbecco’s Modified Eagle Medium/Nutrient Mixture F-12, DMEM/F12), supplemented with 10% foetal bovine serum (FBS), 100 units mL^−1^ penicillin, and 100 μg mL^−1^ streptomycin in a 5% CO_2_ atmosphere chamber at 37 °C. A549 cells (2 × 10^6^ cells well^−1^) were seeded in Petri dishes (100 mm diameter) and kept overnight for attachment. The three PUAs were dissolved in dimethyl sulfoxide (DMSO, final concentration 0.5%) and used for treatments.

Cell morphology was analysed with the acridine orange and ethidium bromide double staining test. Treated A549 cells (5 μM of the three PUAs for 48 h) were collected by centrifugation at 500× *g* for 5 min and changes in cell morphology and staining were identified through comparison with untreated A549. Cells were dyed with 25 μL of a mix composed by 100 μg mL^−1^ of acridine orange and 100 μg mL^−1^ of ethidium bromide (prepared in phosphate-buffered saline (PBS)). Dyed cells (10 μL) were placed on a microscope slide and observed under a confocal microscope (Zeiss, Oberkochen, Germany, LSM510, laser 488 with LP505 filter for green fluorescence; laser 543 with LP 560 filter for red fluorescence) with 25× objective. Green fluoresce was emitted by acridine orange intercalated into normal double-stranded nucleic acids and red fluorescence by acridine orange bound with damaged single-stranded nucleic acids. Moreover, red fluorescence was generated by ethidium bromide penetrated in dead cells with damaged membranes.

Seventy percent confluent cells were treated with 5 μM of decadienal, heptadienal, and octadienal, which induces moderated mortality (higher concentrations showed too severe death effect), as reported by Sansone et al. [29]. Cells were treated for 2 h, since all death genes were significantly expressed [29]. After treatments (three biological replicates were performed), cells were rinsed with phosphate-buffered saline (PBS). A549 were lysed with 1 mL of TRIsure reagent (Bioline, cat. no. BIO-38032). RNA was isolated according to the manufacturer’s protocol. gDNA was eliminated by using a DNase RNase-free kit (Roche, cat no. 4716728001) for each sample, according to the manufacturer’s instructions. Nanoprop was used for the assessment of RNA concentration.

Reverse transcription of RNA (200 ng) was performed using the RT^2^ first strand kit (Qiagen, Hilden, Germany, cat. no. 330401) according to the manufacturer’s instructions. The qPCR experiments were performed in triplicate using the RT^2^ Profiler PCR Array kit (Qiagen, the list of genes analysed are in Table 4). Amplification reactions (10 μL final volume) were run on a ViiA7 (Applied Biosystems). Cycling conditions used were set up in three stages: the first stage at 50 °C for 2 min and 95 °C for 10 min; the second stage at 95 °C for 15 s and 60 °C for 1 min for 40 cycles; the last stage (melt curve) at 95 °C for 15 s, 60 °C for 1 min, and 95 °C for 15 s. qPCR data (Ct-values) were analysed with PCR array data analysis online software (https://geneglobe.qiagen.com/it/analyze/, Qiagen). Reference genes for qPCR of A549 were actin-beta (ACTB), beta-2-microglobulin (B2M), glyceraldehyde-3-phosphate dehydrogenase (GAPGH), hypoxanthine phosphoribosyl transferase 1 (HPRT1), and ribosomal protein large P0 (RPLP0), the expression of which remained constant.

### 4.5. Statistical Analysis

Sea urchin embryos, but also in vitro human cells, possess high intrinsic biological variability; for this reason, gene expression data were analysed with PAST3 software. In particular, two-tailed (Wilcoxon) Mann–Whitney *U* test was used to test whether the medians of two independent samples were different [65,66]. In this way, variability in control groups has been analysed, following assessment of significance for the reported changes in treatment groups.

## Figures and Tables

**Table 1:** Percentage of *Paracentrotus lividus* embryos with normal and abnormal development after the three polyunsaturated aldehyde (PUA) treatments. Results were obtained by morphological analysis at 48 hpf; at least 200 pluteus larvae were examined.

Compound	Concentration (μM)	% Normal (±SD)	% Abnormal (±SD)
Decadienal	Control	94.6 ± 0.6	5.4 ± 0.6
	1.0	85.3 ± 0.8	14.7 ± 0.8
	1.3	74.7 ± 1.4	25.3 ± 1.4
	1.6	61.1 ± 2.1	38.9 ± 2.1
	2.0	34.3 ± 5.2	65.7 ± 5.2
	2.3	25.1 ± 3.9	74.9 ± 3.9
Heptadienal	Control	96.2 ± 1.2	3.8 ± 1.2
	2.0	84.4 ± 1.5	15.6 ± 1.5
	2.5	76.2 ± 1.3	23.8 ± 1.3
	3.0	62.5 ± 1.3	37.5 ± 1.3
	5.5	38.7 ± 4.3	61.3 ± 4.3
	6.0	26.2 ± 2.5	73.8 ± 2.5
Octadienal	Control	95.3 ± 0.9	4.7 ± 0.9
	2.0	87.9 ± 1.4	12.1 ± 1.4
	4.0	76.7 ± 1.2	23.3 ± 1.2
	4.5	63.8 ± 3.0	36.2 ± 3.0
	7.0	39.3 ± 3.7	60.7 ± 3.7
	8.0	23.2 ± 2.9	76.8 ± 2.9

**Table 2:** Up- and downregulation of genes studied in sea urchin *Paracentrotus lividus* embryos (at the highest concentration, at 48 hpf) and A549 cells (at 5 μM, for 2 h). n.s. indicates non-significant gene expression.

Genes	Decadienal		Heptadienal		Octadienal	
	Embryos	A549	Embryos	A549	Embryos	A549
*Ripk*	UP	UP	UP	UP	n.s.	UP
*Tnfr16*	UP	UP	UP	UP	n.s.	UP
*Tnfr19/27*	UP	UP	UP	UP	n.s.	n.s.
*NF-kB*	n.s.	UP	UP	UP	n.s.	n.s.
*Bax*	n.s.	n.s.	n.s.	n.s.	n.s.	n.s.
*Bcl2*	n.s.	UP	n.s.	UP	n.s.	n.s.
*Parp*	n.s.	n.s.	UP	UP	n.s.	n.s.
*Aifm1*	n.s.	n.s.	UP	UP	n.s.	n.s.
*Ulk1/2*	n.s.	n.s.	UP	UP	n.s.	n.s.
*Ulk3*	n.s.	n.s.	UP	UP	n.s.	n.s.
*Pink*	n.s.	n.s.	UP	n.s.	n.s.	n.s.

**Table 3:** Forward and reverse primers of 11 *Paracentrotus lividus* genes studied by qPCR, with *Z12-1* used as reference (primers for *NF-κB* are from Pinsino et al. [61], all others are from Galasso et al. [28]).

*Aifm1*	F: 5′-TAGTGGCAGTGGGTCTGGAA
	R: 5′-CGCCCTAGCTTGATGTCGTA
*Bax*	F: 5′-CGTATCGAGCAGACACGGTT
	R: 5′-GCTGGAAACGCTCCACAATG
*Bcl2*	F: 5′-TAGGGGTATAGCGGCAGTCA
	R: 5′-GGCATCCCATCCTCCTTGTT
*NF-κB*	F: 5′-TCCCATGGAGGACTGCCGTGTCA
	R: 5′-TCGTTGGTTACCAAGGAGACCACA
*Parp*	F: 5′-CCAAGAACCCAATCAAACGCC
	R: 5′-CCTGCACGTTCTTTACTAG
*Pink*	F: 5′-GCAGTTGGTTACCTTGGC
	R: 5′-CGCAATGAAATCGCACATCC
*Ripk*	F: 5′-GGAGGCTCTTTTGGAGACG
	R: 5′-CGATGAACTCAGACGTGAGG
*Tnfr16*	F: 5′-TGGAACCTACTCGGATCTCGT
	R: 5′-CATTGGCTGGTTGGGAAGTC
*Tnfr19/27*	F: 5′-CAACTGAAGAGCCTTCTCC
	R: 5′-GCGTTGTACTGAGCTTGATC
*Ulk1/2*	F: 5′-TTGAAGGCTAGGACACTGGA
	R: 5′-ACTGGCATTGGGGAAGTTGAG
*Ulk3*	F: 5′-GTAATGGAAGCTGTGAAGGC
	R: 5′-CTCTCCTCATGTACTCTAGGC
*Z12-1*	F: 5′-AGCGCCACACCAAAAGAAGTC
	R: 5′-GGATGATAGACAGGGCTGTTTGGA

**Table 4:** List of human genes with accession numbers in UniGene and GenBank (from https://geneglobe.qiagen.com/product-groups/rt2-profiler-pcr-arrays), and short description of encoded proteins.

Names	UniGene	GenBank	Description
*Aifm1*	Hs.424932	NM_004208	Apoptosis-inducing factor, mitochondrion-associated, 1
*Bax*	Hs.624291	NM_004324	BCL2-associated X protein
*Bcl2*	Hs.150749	NM_000633	B-cell CLL/lymphoma 2
*NF-κB*	Hs.654408	NM_003998	Nuclear factor kappa B
*Parp*	Hs.177766	NM_001618	Poly (ADP-ribose) polymerase 1
*Pink*	Hs.389171	NM_032409	PTEN induced putative kinase 1
*Ripk*	Hs.103755	NM_003821	Receptor-interacting serine-threonine kinase
*Tnfr1*	Hs.279594	NM_001065	Tumour necrosis factor receptor superfamily, member 1
*Tnfr2*	Hs.256278	NM_001066	Tumour necrosis factor receptor superfamily, member 2
*Ulk1*	Hs.47061	NM_003565	Unc-51-like kinase 1
*Ulk3*	Hs.168762	NM_014683	Unc-51-like kinase 3

## Supplementary Materials

Supplementary materials can be found at https://www.mdpi.com/1422-0067/21/15/5201/s1. Figure S1: Abnormalities induced at 48 hpf by the three PUAs in sea urchin *Paracentrotus lividus* embryos; Figure S2: Abnormalities and delays induced at 5 and 21 hpf by the three PUAs in sea urchin *Paracentrotus lividus* embryos; Figure S3: Cell death induced after 48 h by the three PUAs on human adenocarcinoma cells (A549); Figure S4: Cycle threshold (Ct) values for the reference gene *Pl_Z12-1* in all experimental conditions.
