# Supplementary material for: Diatom-Derived Polyunsaturated Aldehydes Activate Similar Cell Death Genes in Two Different Systems: Sea Urchin Embryos and Human Cells
Source: Int J Mol Sci. 2020 Jul 22;21(15):5201. doi: 10.3390/ijms21155201 (PMC7439121; doi:10.3390/ijms21155201)
Supplement: Supplementary file 1 [file ijms-21-05201-s001.pdf]

International Journal of
Molecular Sciences

MDPI

Supplementary Materials

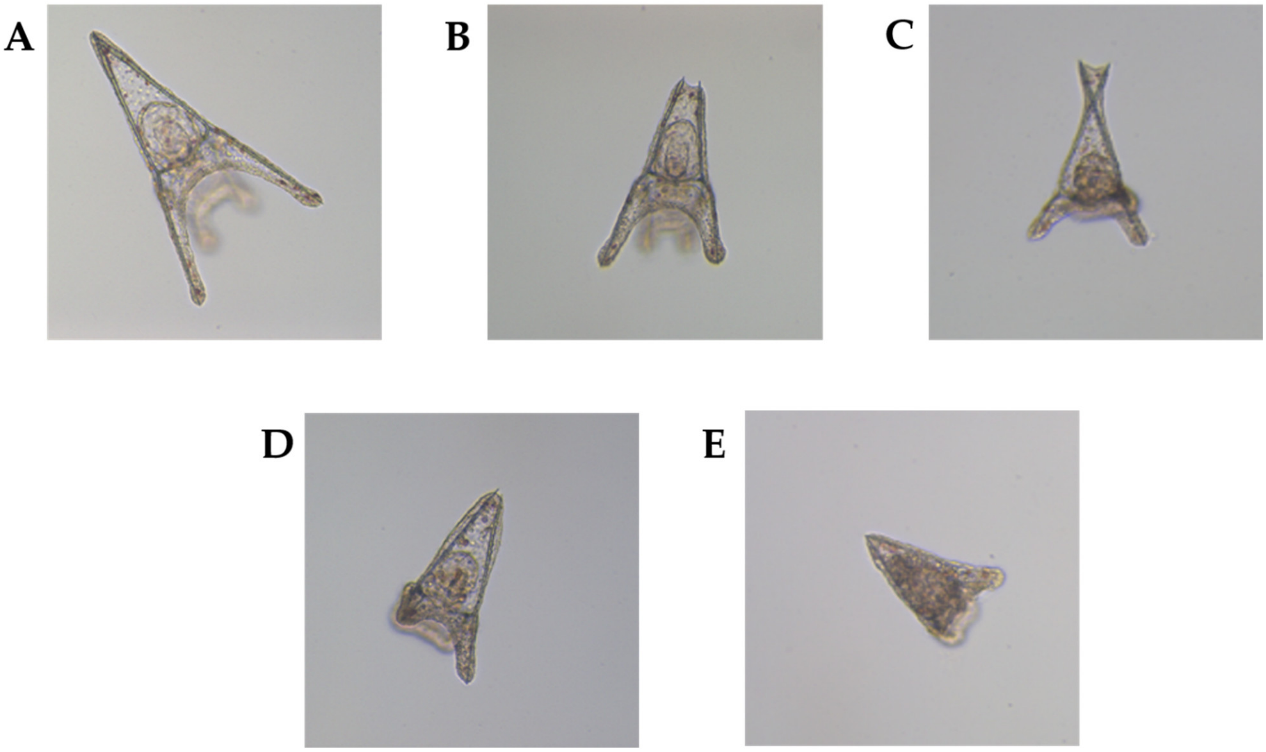

Figure S1. Abnormalities induced at 48 hpf by the highest concentrations of the three PUAs in sea urchin *Paracentrotus lividus* development. Plutei were observed using an inverted microscope (Zeiss Axiovert 135TV) and images were obtained using a Zeiss Axiocam connected directly to the microscope. (**A**) Control, pluteus normally developed; (**B**) pluteus with separated tips; (**C**) pluteus with crossed tips; (**D**) pluteus with not fully developed arms; (**E**) pluteus with incomplete skeletal rods. All developmental perturbations and delays were grouped and reported as % of abnormalities (Table 1).

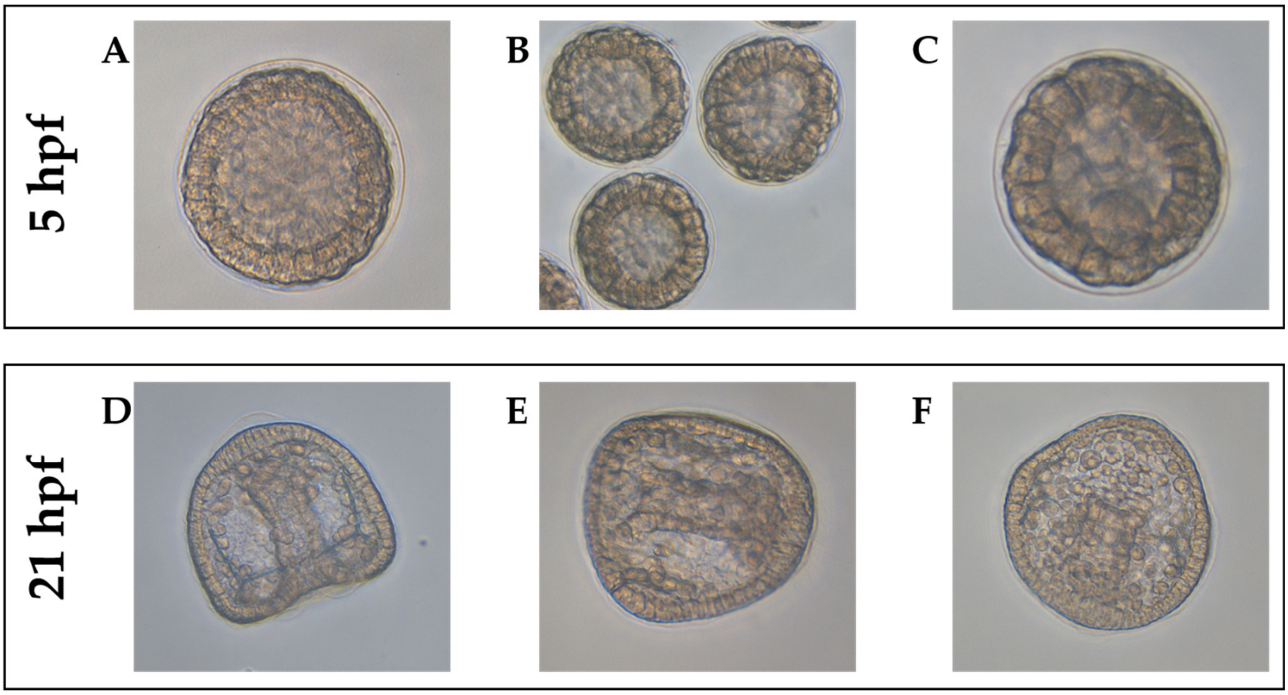

Figure S2. Abnormalities and delays induced at 5 and 21 hpf by intermediate and highest concentrations of the three PUAs in sea urchin *Paracentrotus lividus* embryos. Plutei were observed using an inverted microscope (Zeiss Axiovert 135TV) and images were obtained using a Zeiss Axiocam connected directly to the microscope. (**A**) and (**D**) are control embryos at 5 and 21 hpf; (**B**) and (**E**) are abnormal/delayed embryos at 5 and 21 hpf treated with intermediate doses; (**C**) and (**F**) are abnormal/delayed embryos at 5 and 21 hpf treated with highest doses.

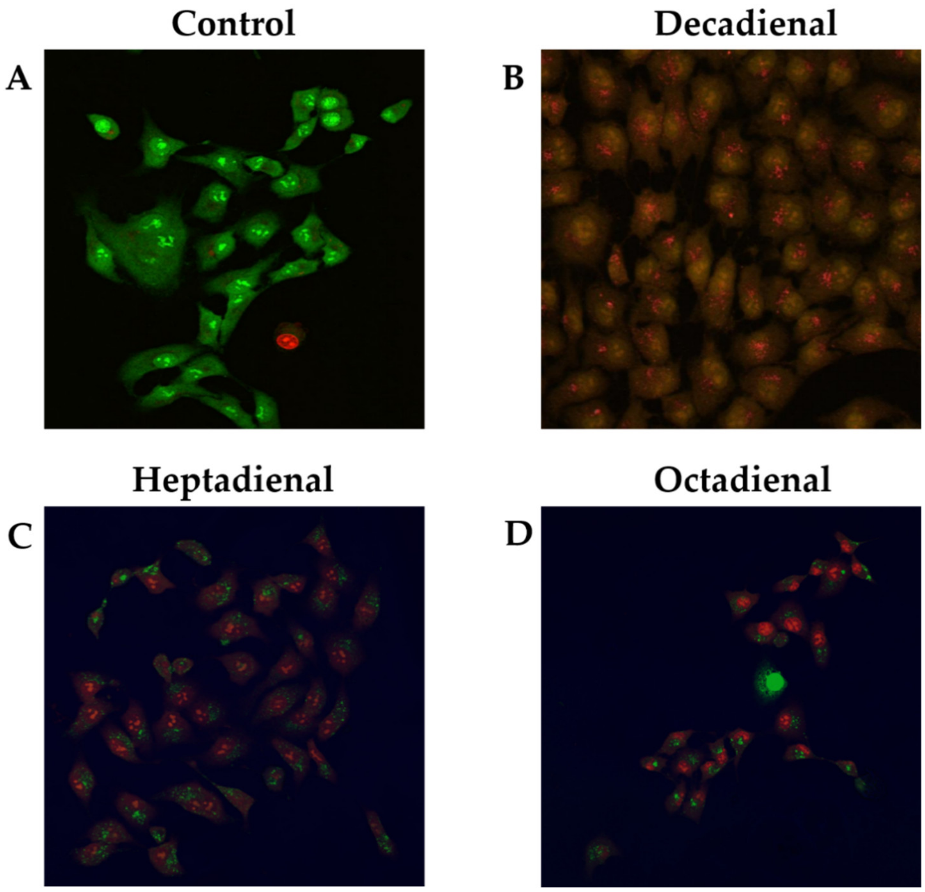

Figure S3. Cell death induced after 48 h by 5 μM of decadienal (**B**), heptadienal (**C**) and octadienal (**D**) on human adenocarcinoma cells (A549). Cells were observed and compared to the control (untreated A549, **A**) using a confocal microscope (Zeiss LSM510, laser 488 with LP505 filter for green fluorescence; laser 543 with LP 560 filter for red fluorescence) with 25x objective. Green fluorescence indicates penetration of acridine orange in living cells, which is intercalated into intact double-strains nucleic acids. Red fluorescence indicates penetration of acridine orange in living and dying cells, which is intercalated into broken single strand nucleic acids; same fluorescence is due to penetration of ethidium bromide into dying cells with damage membranes.

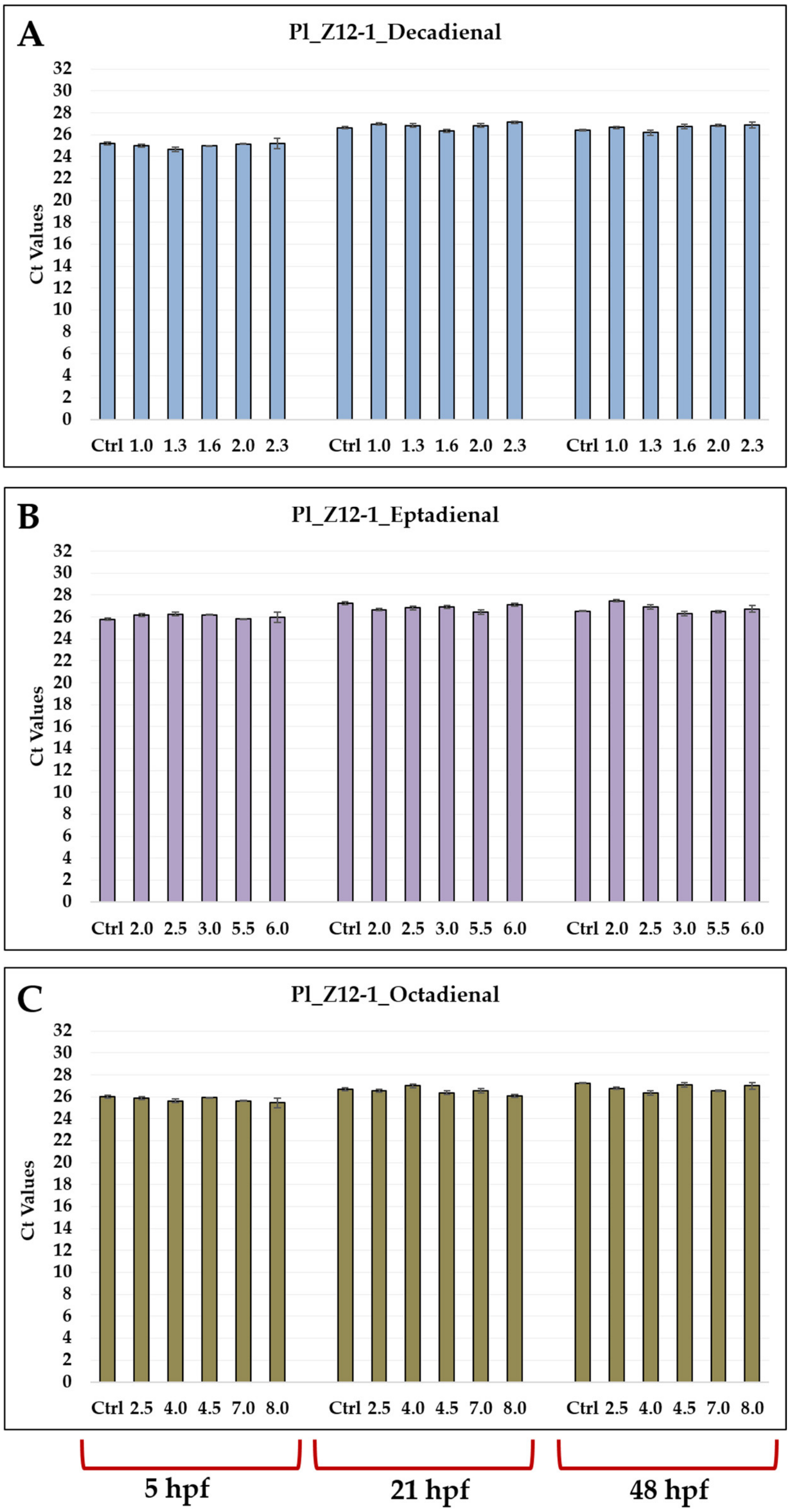

Figure S4. Cycle threshold (Ct) values for the reference gene *Pl_Z12-1* in all experimental conditions. Ct values have been monitored during normal sea urchin embryos development (at 5, 21 and 48 hpf, see control values in figure **A**, **B** and **C**) and after 5, 21 and 48 hpf of treatments at five concentrations of decadienal (**A**), heptadienal (**B**) and octadienal (**C**). Values are reported for controls and treatments as mean ± standard deviation of three biological replicates.
